# On the Elephantiasis Græcorum Endemic in Certain Parts of Norway

**Published:** 1844-09-07

**Authors:** D. C. Danielssen


### ON THE ELEPHANTIASIS GRÆCORUM ENDEMIC IN CERTAIN PARTS OF NORWAY.

By D. C. Danielssen, M. D.

Greek leprosy, once so common among the Israelites, and which in the middle ages was endemic over the whole of Europe, and appears to have been particularly common in Great Britain, prevails at this moment in Norway, probably with as much severity as ever. In a population of 200,000, 1200 are actually lepers.

Greek leprosy presents itself especially along that portion of the western coast of Norway which is comprised between the 60th and 70th degrees of North latitude, among the poorer classes of the community: it is hereditary, but does not attack every member of the family when one is invaded; it is not contagious, but its severity appears to increase with the number of generations which have suffered from it. Its invasion is determined by accidental external circumstances. No age gives immunity; that form which presents the tuberculous character is evolved in the fœtus, and has been seen in a new-born infant. It may arise in a healthy individual, born of healthy parents, living under the influence of the conditions which favour its evolution; these conditions being especially damp and dirty clothes, small and ill ventilated houses, exposure to the thick fogs of the country, indifferent food, and the other accompaniments of poverty.

Greek elephantiasis, or leprosy, presents itself under two forms in Norway—El. tuberculosa, and El. anæsthetica, but the two forms occur together, and also complicate other diseases of the skin, such as eczema, prurigo, lichen, &c.

In the bodies of those who fall victims to the disease—and its tendency is invariably towards a fatal termination—hard, yellowish, and granulated masses are found in the substance of the dermis, and in the subjacent cellular tissue, which destroy the structure of these textures. The same alteration occurs in the greater number of organs—in the parietes of the subcutaneous veins, in the coats of the eye, the larynx, trachea and bronchi, pleura, liver, spleen, intestinal canal from top to bottom, and the uterus: strangely enough, the substance of the lungs is generally exempt. In a few cases of El. anæsthetica, the skin in several places was found very much atrophied, the subcutaneous cellular tissue and the muscles almost entirely destroyed, and the tendons in some places retracted.—*Ibid.*

